# Experimental and theoretical studies of nonlinear dependence of the internal resistance and electrode thickness for high performance supercapacitor

**DOI:** 10.1038/srep45934

**Published:** 2017-04-05

**Authors:** Xilong Liu, Xiaohang Dai, Guodong Wei, Yunlong Xi, Mingjun Pang, Volodymyr Izotov, Nickolai Klyui, Dmytro Havrykov, Yuan Ji, Qing Guo, Wei Han

**Affiliations:** 1 Key Laboratory of Physics and Technology for Advanced Batteries, Ministry of Education, College of Physics, Jilin University, Changchun 130012, China; 2 Institute of Carbon Materials Science, Shanxi Datong University, Datong 037009, China; 3 V. Lashkaryov Institute of Semiconductor Physics, National Academy of Sciences of Ukraine, 41 Nauki Pr., 03028 Kyiv, Ukraine

## Abstract

In this study, the internal resistance with the increasing of electrode thickness in a typical nanoporous carbon-based supercapacitor and their corresponding electrochemical performances were designed and investigated in detail. As for the carbon-based double electrode layer electrochemical system, electrochemical experiments greatly support the fact of nonlinear dependence and indicate that the curve of internal resistance vs. electrode thickness can have a minimum value when the thickness increasing from 10 to 140 μm. To explain the underlying mechanisms responsible for the nonlinear dependence, a theoretical model based on a porous electrode/electrolyte electrochemical system was proposed. As expected, the results of calculations carried out in the framework of the proposed model can be very good agreement with the experimental data. According to the calculation, the optimized electrode thickness is 53.1 μm corresponding to the minimum value of SC internal resistance. Obviously, the current research results might greatly support the nonlinear conclusion instead of linear relationship between the internal resistance and the electrode thickness and may shed some light on the fabrication and exploration of supercapacitors with high power density.

An intense development of high performance electric vehicles (especially electric cars) requires novel rechargeable power sources that could endure up to one million charging/discharging cycles as well as high specific power and high specific energy^1^. Up to date, though several kinds of energy storage devices have been developed for the hybrid electric, it is widely accepted that the electric double-layer capacitors, also known as the supercapacitors (SC) is an ideal choice due to the long durable life with practically unlimited charging/discharging cycles^2^. A typical SC device can be usually consisted of two carbon-based electrodes that are electrolyte-impregnated and separated with a separator, whose structure is similar with that of a common electrolytic capacitor. Yet different from the electrodes of the common electrolytic capacitors, a SC electrode consists of a metal collector coated with a layer of nanoporous carbon material with high specific surface area^2^.

A key parameter to determine the SC performance is the specific energy density, and for this reason, there is considerable interest in enhancing their values by optimizing SC electrode structure and design. The specific energy density for commercial SC devices can reach up to 6 W · h/kg from the key firms’ products^3^. It is also well known that specific energy value can be mostly related to the fabricated SC electrodes. According to the energy store mechanism, in the course of SC charging, the energy can be accumulation of electric charge in the double layer formed at the carbon electrode/electrolyte interface^2^. Therefore, the specific capacity (based on the energy density formula E = 0.5 CV^2^, in fact, the energy density is only related to the specific capacity if the voltage is fixed at a certain value) for a typical carbon-based SC with organic electrolyte can be about 10 μF/cm^2^,^3^,^4^. The specific capacity can be influenced by the parameters of the conductivity, surface area and pore size distribution of carbon material, binder content, electrode thickness, and *et al*. Among these parameters, large specific surface area (over 1000 m^2^/g) of porous carbon material used in the SC electrodes can facilitate the achievement of high specific energy density in the final SC device.

Another important parameter to determine the SC performance is the internal resistance (R_in_). The R_in_ value determines another important SC parameter that generally called power density, namely, its specific pulse power *P*(W/kg) that can be calculated from the following equation:


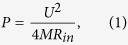


where *U*(V) and *M*(kg) are the SC operating voltage and mass, respectively. For products from the leading manufacturers, *P* value ranges from 10 kW/kg to 20 kW/kg^3^. Based on Eq. (1), higher *P* values can be achieved by decreasing the R_in_ value. According to the reports in the literature, the R_in_ depends on the following factors: (i) the contact resistance between the metal Al current collector and the electrode component made of a porous carbon material; (ii) resistance resulted from the interelectrode region; (iii) resistance from the electrode component and soaked with electrolyte. From here on, such the electrode components will be referred to as the energy-storage electrode components.

It is widely accepted that the main contribution to the SC R_in_ comes from the resistance of the energy-storage electrode component. According to reports, it can depend on many factors: (i) size of pores of the activated carbon material, (ii) composition of the material used for production of the energy-storage electrode component, (iii) electrolyte composition, and (iv) thickness of the energy-storage electrode component. To reduce the SC R_in_ value, several cost-effective and simple methods were explored for solving this challenge from the industry technological views^5^,^6^,^7^,^8^. Though decades of sustained efforts, it is still a very hot study field and still need substantial progress on developing new technologies for reducing the R_in_. Among these mentioned factors, the resistance resulted from the thickness of the energy-storage electrode component can have a main influence on the SC performance for the industry products. For the purpose of practical application, high mass loading of active materials with thick electrode for the fabrication of supercapacitors can achieve high energy density. However, the increase in the electrode thickness usually leads to increasing resistance as well as reducing specific capacitance and energy density, due to close-packed structures resulting in a limited electrochemically active surface area. Moreover, the resistance dependence of electrode thickness is not clearly and even inconsistent in the literature reports. Some reports support this dependence is linear^9^,^10^,^11^, while other ones believe that the functional dependence of SC internal resistance on electrode thickness is nonlinear^12^,^13^. Therefore, experiment and theory study should be performed to discover the underlying mechanism to increase the utilization efficiency of electroactive material at large mass loading with optimized electrode thickness. In addition, application of the electric-spark method for processing of a metal current collector by implanting carbon particles into its surface leads to considerable decrease of contact resistance between the current collector and energy-storage electrode component, which could be regarded as a promising method for practical application. The detailed study of the theory and performance of the current collector modification could be found in our group report in the near future.

In this study, the resistance dependence of the electrode thickness and their corresponding electrochemistry performance were investigated in detail. The nonlinear results for SC R_in_ dependent of electrode thickness in a typical carbon-based two double layer SC can effectively eliminate the arguments^9^,^10^,^11^,^12^,^13^. More importantly, a theory model based on electrochemical system “porous carbon material/organic electrolyte” was proposed for explaining the nonlinear dependence fact. By tuning the electrode thickness alone over a wide range from 10 to 140 μm in the course of experiments (the rest of system parameters remaining constant), it is found that the theory calculations can agree well with the experimental results.

## Methods

### Raw materials

All the reagents such as 1-Methyl-2-pyrrolidinone (NMP), carbon super P, and polyvinylidene fluoride (PVDF) used in this study were of analytical grade and were used as received without any further purification The used activated carbon powders were purchased from “SHIHEZI SETDTIANFU TECHNICAL” (China) and before use, let them pass through a sieve with pore diameter of 10 μm.

### Preparation of the electrodes

The electrode slurry for preparation of energy-storage electrodes were using a mixed suspension solution containing with NMP (solvent), activated carbon powders, Carbon Super P as a conducting additive, PVDF served as a binder, followed mixing these materials by means of ultrasonic treatment. The weight ratio between NMP, activated carbon material, conductive additive and PVDF was fixed at 75: 22.5: 1.25: 1.25. Subsequently, the electrodes were made by deposition of suspension layers using a “Doctor Blade” installation onto a modified aluminum foil. The electrodes were then dried for 72 h in a vacuum oven (pressure of 1 kPa) at a temperature of 150 °C to remove NMP from the carbon-bearing electrode components.

### Structure characterizations

The activated carbon powders used in this research were characterized with field emission scanning electron microscopy (FESEM, S-4800, Hitachi, Japan), X-ray diffraction (XRD, D8 ADVANCE, Bruker, German) and Transmission electron microscope (TEM, JEM-2100F, JEOL, Japan). The pore size distribution for that powder was determined using nitrogen adsorption-desorption at a temperature of 77 K on a specific surface area and porosity analyzer (ASAP 2020HD88, Micromeritics, USA).

### Electrochemical measurements

The electrochemical characteristics of the electrodes were examined using typical SC device models containing 1.3 М of Et_4_NBF_4_ solution in acetonitrile as the electrolyte. Cyclic voltammeter (CV) tests were performed at the applied voltages of 0–2.5 V, Galvanostatic charge-discharge (GCD) tests were performed at 100 mA, and impedance spectroscopy in the frequency range from 10 kHz down to 0.01 Hz by using a bipotentiostat (760E, China). All the electrochemical tests were performed at room temperature.

## Results

**Figure 1:**
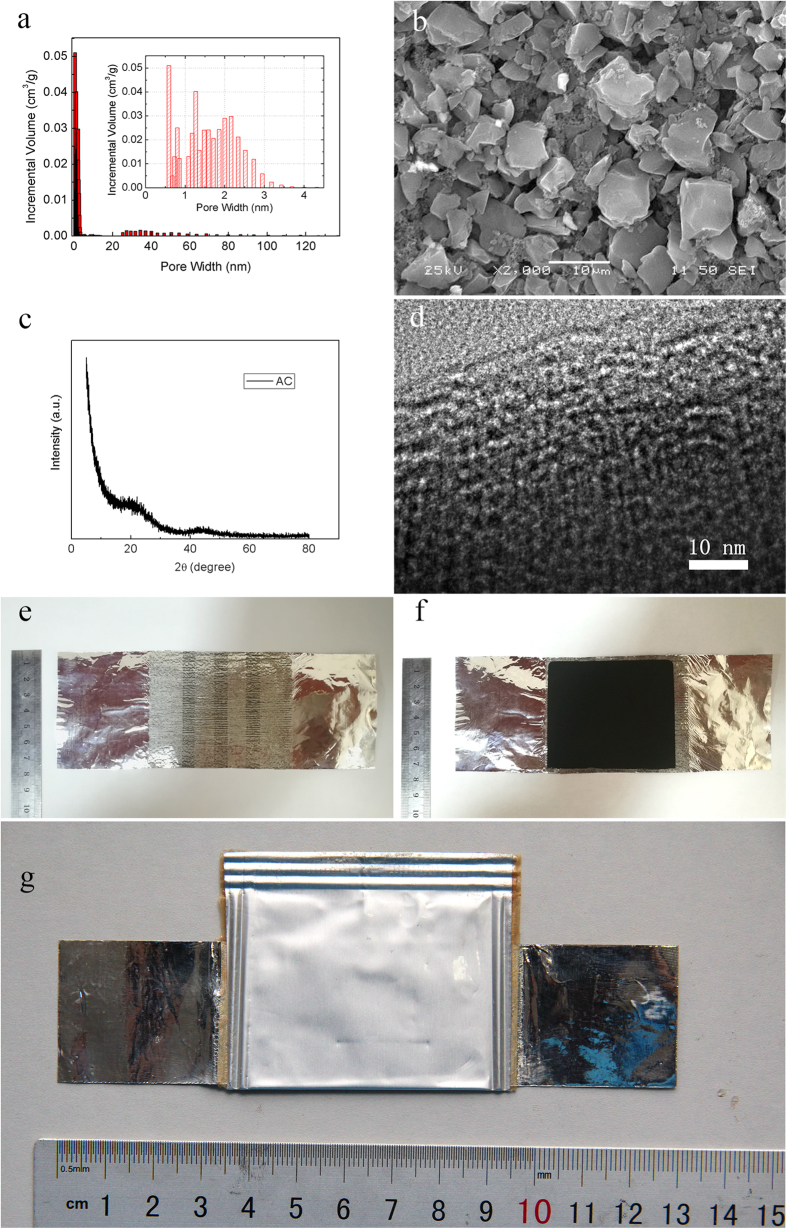
(**a**) The pore size distribution and (**b**) a typical SEM image of activated carbon powders, respectively. The inset in (**a**) is the pore size distribution curve with enlarging the X-axis in the range of 0–4 nm. (**c**) XRD pattern of porous carbon powder and (**d**) the mesoporous on the activated carbon powders surface, respectively. Photo images of (**e**) aluminum foil modified by the electric-spark method, (**f**) the electrode sheet with coating electrode slurry, and (**g**) the final sealed SC device model, respectively.

As shown in Fig. 1a, according to the Barrett–Joyner–Halenda (BJH) pore size distribution analysis determined from the adsorption branches, the as-used activated carbon powders contain micro-, meso- and macropores with a BET surface area of ~1900 m^2^/g. From the inset in Fig. 1a, these pores are most micro- and mesopores with the diameter up to 3 nm, which mainly contribute to the surface area value. Figure 1b presents the morphology and microstructures of the used activated carbon powders. The powders are consisted of irregular particles with the size up to 10 μm. Figure 1c is the XRD pattern of used porous carbon powder, indicating the amorphous nature. From the HRTEM image as displayed in Fig. 1d, the mesoporous of the activated carbon can be clearly observed with the pore size distibution around 2 nm. When used for fabricating electrode, these particles can be combined to form paper-like electrode sheet with desired thickness by the binder. The biomass-derived activated carbon powders are very loosely packed and contained many micro- and mesopores in a single particle body. Obviously, micro- and mesopores with size less than 3 nm can contribute a significant portion of pore volume in the particle according to our bet measurements. Due to smaller micro pores (<2 nm) are more difficult to access during the SC charging-discharging cycles, these pores (>2 nm) can serve as transport channels that pass from the metal collector through the energy-storage electrode component to the electrode surface. The electrodes were prepared by coating slurry with different thicknesses varied from 14 μm to 133 μm using a “Doctor Blade” installation onto the aluminum foil (size: 3 cm*3 cm) modified by the electric-spark method^8^ (Fig. 1e). The electrode together with Al foil (Fig. 1f) was dried for 72 h in a vacuum oven (pressure of 1 kPa) at the temperature of 150 °С to remove NMP from the carbon-bearing electrode component. Subsequently, the resulting electrodes were placed into a glovebox under argon atmosphere. The SC device models can be assembled with two electrodes with the same coating thickness and the two electrodes are parted with a separator produced by Nippon Kodoshi Corporation (Japan). After the electrodes and separator were together loaded into a case of 130 μm thick laminated aluminum foil produced by Showa Denko K.K. (Japan), the case was sealed over along its perimeter with a special Showa Denko hot-melt glue. A polypropylene connecting pipe was inserted into a hot-melt glue layer in the course of sealing. After sealing process, 1.3 М of Et_4_NBF_4_ solution in acetonitrile (as electrolyte) was injected into the SC models through the connecting pipe. Finally, the SC device models (Fig. 1g) were fabricated after the connecting pipe was taken away.

**Figure 2:**
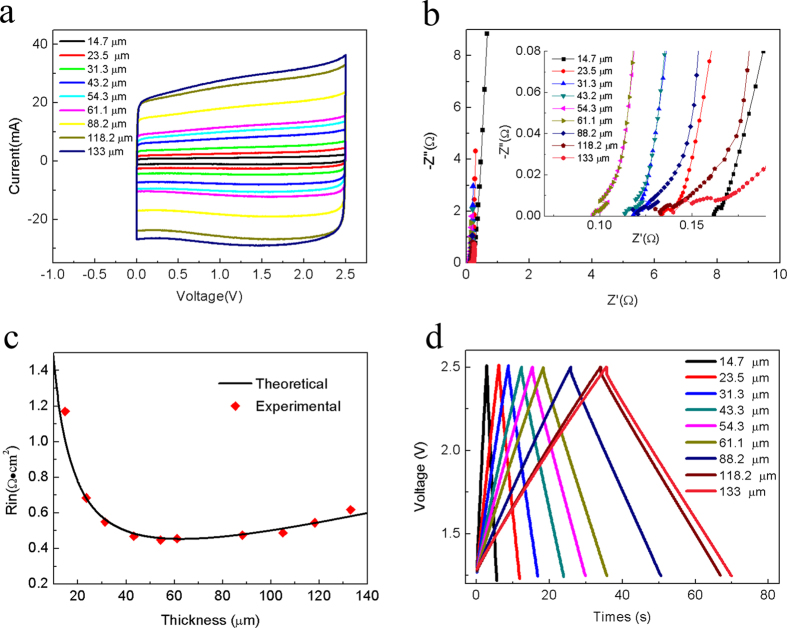
(**a**) CV curves measured at the potential scanning speed of 5 mV/s for the SC models and (**b**) their corresponding impedance hodographs for SC models with different electrode thicknesses in the range of 10–140 μm. (**c**) Experimental data and theoretical curve of the dependence of internal resistance on the thickness of the electrodes. (**d**) The GCD curves of 100 mA for the SC models

Electrochemical performances of the SC models with the electrode thickness in the range of 10–140 μm were studied by using CV within a 0.0–2.5 V range at potential scanning speed of 5 mV/s and GCD with 100 mA current, impedance spectroscopy was performed with the frequency range from 10 kHz down to 0.01 Hz, respectively. All the electrochemical measurements were performed at room temperature. As shown in Fig. 2a, the shape of all the CV profiles is nearly the ideal rectangular shapes, indicative of highly capacitive behavior with good ion response. No obvious current density peaks and drastic growth in all the measured CV curves, indicating no pseudo capacitive capacitance contribution and absence of unwanted electrochemically active impurities in that potential range. The form of ideal rectangular CV curves enables one to state that only adsorption and desorption of cations Et_4_N^+^ and anions BF_4_^−^ occur at the electrode surface in SC models over a given potential range. The SC parameters can be determined using impedance spectroscopy displayed in Fig. 2b. The R_in_ of SC model that corresponded to the position of experimental point on the real *Z’* axis of the impedance curve can be determined at the maximal frequency of 10 kHz. Therefore, the R_in_ measurement values with changed electrode thickness can be obtained presented in Fig. 2c.

## Discussion

**Figure 3:**
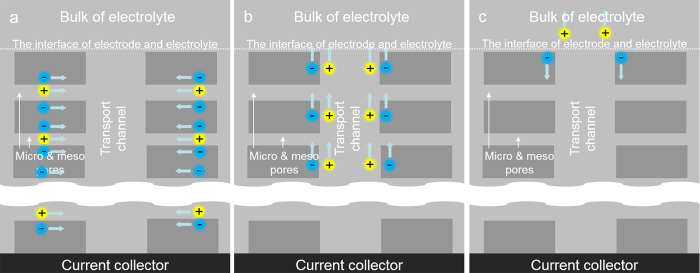
The schematic diagram of the desorbed ions’ diffusion.

Obviously, from Fig. 2c the dependence of internal resistance on electrode thickness is nonlinear instead of linear for the electrochemical system under investigation. Therefore, a possible mechanism of electrochemical processes proceeding in SC should explain the above nonlinear dependence fact. As stated above, the energy-storage electrode component consists of porous activated carbon structures with rich of micro-, meso- and macropores and these pores with the surface area of ~1900 m^2^/g can be serves as transport channels for electrolyte ions. When charging the SC, the electrolyte ions can penetrate into the energy-storage electrode component through these transport channels and can be adsorbed on the pore surface of carbon material particles. In the course of SC discharging, the desorbed ions can diffuse away from the surface adsorbed sites of the energy-storage electrode component. The diffuse journey for desorbed ions can be divided as followed steps (shown in Fig. 3): (1) Firstly, the ions can diffuse through pores of the carbon material into the transport channels. The charge induced by ions at the carbon material surface can move parallel to the positive ion diffusion. (2) At the second stage, the ions can move through the transport channels towards the electrode surface and at the same time the induced charge can move parallel to the positive ion movement. (3) At the final stage, the ions enter into the electrolyte bulk, while the induced charge goes through the energy-storage electrode component to the metal collector and then comes to the external electrical circuit.

To build a model for description of the above mechanism of SC discharging, an assumption that the pores of activated carbon material as well as transport channels are uniformly distributed over the whole volume of the energy-storage electrode component. The total thickness of the energy-storage part of electrode as the model is *h*. The model parameters can be determined from the experimental data and all the parameters can be calculated by using the ideal single layer electrode sheet with per 1 cm^2^ of visible surface. Resistivity of the transport channels and the energy-storage electrode component can be determined from the piece of experimental curve R_in_(h). The dependence of internal resistance on *h* is described by the straight-line equation:


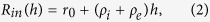


where *ρ*_*i*_ (Ω · cm^2^/μm) is the resistivity of transport channels, *ρ*_*e*_ (Ω · cm^2^/μm) is the resistivity of the energy-storage electrode component, respectively. Here *r*_0_ (Ω · cm^2^) is the total resistance of the separator (soaked with electrolyte) and the transition layer between the energy-storage electrode component and metal current collector. The latter is determined from the following equation:


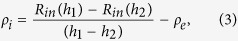


where *R*_*in*_(*h*) (Ω · cm^2^) is the internal resistance of SC model corresponding to the thickness *h. ρ*_*n*_ (Ω · μm · cm^2^) is the resistivity of nanopores carbon material of the energy-storage electrode component. Therefore, *ρ*_*n*_ as the ionic resistance of carbon material placed in a rectangular trapezoid with base area *S* = 1 cm^2^ and height *h*_*t*_ = 1 μm (the base of trapezoid is parallel to the metal collector) can be calculated by the following equation:


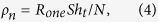


where *N* is the number of carbon material pores in the volume of trapezoid, and *R*_*one*_ (Ω) is the ionic resistance of a single carbon material pore, respectively. According to the most reports and our BET results, in the course of charging for the SC device with the organic electrolyte, most of electric charge can be accumulated only in the mesopores with diameters in the range of 2–3 nm. Therefore, only these pores can keep the charges and release them back at SC discharging process. In fact, when charges travel from a transport channel to a pore whose size is comparable to that of ion, these pores will lose their solvation sheath^14^,^15^ and lead to greatly decrease of diffusion coefficient by several orders of magnitude^16^. Therefore, resistivity resulted from these pores with small diameter can exceed that of a transport channel by several orders of magnitude, and the total resistance of all the micropores can be inversely proportional to the number of those participating in process of discharging.

Based on the above discuss, to further determine *ρ*_*n*_, another model assumption should be made. It is supposed that the ionic component of *R*_*in*_(*h*) within a certain range of *h* values can be determined by that of the carbon material. Thus, the ionic component of *R*_*in*_(*h*) with two different values *h*_1_ and *h*_2_ (*h*_1_ < *h*_2_) can be calculated within the above range by using Eqs (6) and (7) given below. According to the equivalent circuit of the energy-storage electrode component, the ionic component of internal resistance *r*_*ion*_(*h*) (Ω · cm^2^) can be obtained from Eq. (5):


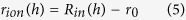



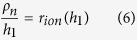



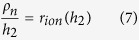


The validity of our assumption can be verified by the experimental results presented in Fig. 2c. In our case, when *h*_1_ = 14.7 μm and *h*_2_ = 23.5 μm, 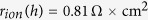 and, 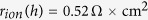 can be obtained, respectively. Therefore*, ρ*_*n*_ can be calculated from the following expression:


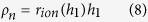


From our model, it is possible to estimate the internal resistance values dependence of energy-storage component thickness h^*^. The minimal can be found at the cross point of the descending branch (described as 
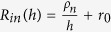
) and ascending branch (described as 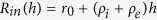). The h^*^ value can be calculated as


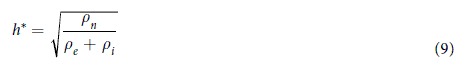


Eq. (9) determines thickness of the energy-storage electrode component corresponding to the minimal of SC internal resistance. Within the framework of the model considered, the equation is as follows, describing how the SC internal resistance depends on electrode thickness:


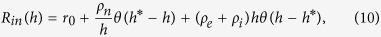


where *θ*(*x*) is the Heaviside step function whose value is 0 if *x* < 0, 0.5 if *x* = 0 and 1 otherwise^17^. The parameters *ρ*_*e*_ + *ρ*_*i*_, *r*_0_ and *ρ*_*n*_ that characterize the considered electrochemical system can be calculated based on the experimental data with Eqs (2), (3) and (8). To determine the parameters *r*_0_ and *ρ*_*e*_ + *ρ*_*i*_, th_*e*_ points lying along the straight portion of the experimental curve *R*_*in*_(*h*) are chosen. In the set of experiments, using the values of SC internal resistance corresponding to the electrode thickness values with 133 μm, 118.2 μm, 88.2 μm and 61.2 μm, respectively, *r*_0_ = 0.68 Ω · cm^2^ and *ρ*_*e*_ + *ρ*_*i*_ = 0.0043 Ω · cm^2^/μm can be obtained.

The parameter *ρ*_*n*_ can be determined by using these points lying along the experimental *R*_*in*_(*h*) curve for which the product *r*_*ion*_*h* remains the same. From Table 1, these requirements can be met with the electrodes thickness of *h*_1_ = 14.7 μm (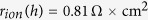), *h*_2_ = 23.5 μm (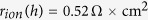) and 31.3 μm, (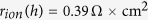) thick, respectively. For SC models with such electrodes, the products *r*_*ion*_*h* are in good agreement with the experimental results within the experimental error, indicating the validity of the theoretical model proposed. The mean value of *ρ*_*n*_ calculated using the experimental data for the above points is 12.13 Ω · μm · cm^2^. Using the above *ρ*_*e*_+ and *ρ*_*n*_ values, the optimized electrode thickness is h^*^ = 53.1 μm corresponding to the smallest R_in_ value.

## Conclusions

Based on the experimental studies as well as the correlation between the data obtained and calculated parameters of the prepared SC electrochemical system, the conclusions can be obtained as following:

1. It is found that the dependence of SC internal resistance on the activated carbon-based electrode thickness is nonlinear.
2. A theoretical model is proposed to explain the nonlinear dependence of the internal resistance and electrode thickness. Obviously, the calculations are in good agreement with the experimental results within the experimental error.
3. When the optimized electrode thickness is 53.1 μm, the SC internal resistance with the minimum value is 12.13 Ω · μm · cm^2^. The advanced approach can be promising for prediction of parameters of composite materials for SC with high specific power.

## Additional Information

**How to cite this article**: Liu, X. *et al*. Experimental and theoretical studies of nonlinear dependence of the internal resistance and electrode thickness for high performance supercapacitor. *Sci. Rep.* **7**, 45934; doi: 10.1038/srep45934 (2017).

**Publisher's note:** Springer Nature remains neutral with regard to jurisdictional claims in published maps and institutional affiliations.

## Figures and Tables

**Table 1:** The model parameters, results of theoretical calculations and experimental data obtained for the SC models studied, respectively.

*h*	*R*_*e*_	*R*_*in*_	*r*_*ion*_	*r*_*ion*_*h*	*R*_*in*_*(theor)*	*R*_*ion*_*(theor),*	C		
μm	Ώ · cm^2^	Ώ · cm^2^	Ώ · cm^2^	Ώ · μm · cm^2^	Ώ · cm^2^	Ώ · cm^2^	F · cm^−3^	F · cm^−2^	F · g^−1^
14.7	0.271	1.49	0.81	11.95	1.53	0.85	31.9	0.05	72.0
23.5	0.325	1.20	0.52	12.30	1.21	0.53	42.6	0.10	101.7
31.3	0.312	1.06	0.39	12.14	1.08	0.40	46.1	0.14	111.2
43.2	0.357	0.99	0.31	13.56	0.97	0.29	48.3	0.21	115.3
54.3	0.385	0.92	0.22	12.13	0.92	0.23	48.0	0.26	117.4
61.1	0.415	0.94	—	—	0.94	—	50.0	0.31	120.9
88.2	0.438	1.06	—	—	1.06	—	50.8	0.45	122.5
118.2	0.505	1.17	—	—	1.18	—	50.6	0.60	122.1
133	0.559	1.26	—	—	1.25	—	47.0	0.63	113.6
